# Interactions among Quorum Sensing Inhibitors

**DOI:** 10.1371/journal.pone.0062254

**Published:** 2013-04-23

**Authors:** Rajat Anand, Navneet Rai, Mukund Thattai

**Affiliations:** 1 National Centre for Biological Sciences, Tata Institute of Fundamental Research, UAS/GKVK Campus, Bangalore, India; 2 Department of BioSciences and Bioengineering, Indian Institute of Technology Bombay, Powai, Mumbai, India; University of Illinois at Urbana-Champaign, United States of America

## Abstract

Many pathogenic bacteria use quorum sensing (QS) systems to regulate the expression of virulence genes in a density-dependent manner. In one widespread QS paradigm the enzyme LuxI generates a small diffusible molecule of the acyl-homoserine lactone (AHL) family; high cell densities lead to high AHL levels; AHL binds the transcription factor LuxR, triggering it to activate gene expression at a virulence promoter. The emergence of antibiotic resistance has generated interest in alternative anti-microbial therapies that target QS. Inhibitors of LuxI and LuxR have been developed and tested in vivo, and can act at various levels: inhibiting the synthesis of AHL by LuxI, competitively or non-competitively inhibiting LuxR, or increasing the turnover of LuxI, LuxR, or AHL. Here use an experimentally validated computational model of LuxI/LuxR QS to study the effects of using inhibitors individually and in combination. The model includes the effect of transcriptional feedback, which generates highly non-linear responses as inhibitor levels are increased. For the ubiquitous LuxI-feedback virulence systems, inhibitors of LuxI are more effective than those of LuxR when used individually. Paradoxically, we find that LuxR competitive inhibitors, either individually or in combination with other inhibitors, can sometimes increase virulence by weakly activating LuxR. For both LuxI-feedback and LuxR-feedback systems, a combination of LuxR non-competitive inhibitors and LuxI inhibitors act multiplicatively over a broad parameter range. In our analysis, this final strategy emerges as the only robust therapeutic option.

## Introduction

High adaptive mutation rates and lateral gene transfer have resulted in the widespread emergence of antibiotic-resistant bacteria [1]–[3]. This has generated renewed interest in alternative anti-microbial strategies [4]–[6]. Antibiotics exert their effects by blocking or inhibiting bacterial growth, which favors the selection of antibiotic resistance [7]. Strategies that target virulence pathways or antibiotic resistance mechanisms such as biofilm formation, while still leaving bacteria viable, would face less stringent selection. Many human pathogens – including *Pseudomonas aeruginosa*, *Vibrio cholerae*, and *Staphylococcus aureus* – express virulence genes and biofilm-formation genes at high cell densities, presumably as an immune-evasion strategy [8]–[11]. This is achieved by a cell-to-cell communication mechanism known as quorum sensing (QS) [12]–[14]. Quorum-sensing inhibitors are therefore promising candidates for anti-microbial therapy [15], [16]. Natural and synthetic QS inhibitors against various molecular targets have been identified [17]–[21] and some have been shown to function in vivo, reducing mortality in animal models of bacterial infection [22]–[25]. However, it is possible for pathogens to evolve resistance even against QS inhibition [26]–[28]. Effective therapy might therefore require multi-drug approaches [29]. In this effort, pharmacological screens and experiments on specific infection models can be complemented by computational studies [30]–[34]. Here we use a molecular-level model of quorum sensing to assess the efficacy of inhibitor combinations in suppressing virulence.

**Figure 1:**
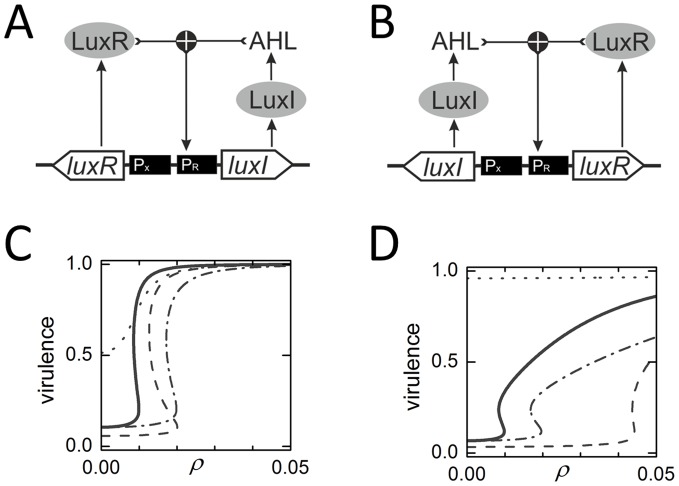
Quorum sensing feedback systems and density-dependent responses. (A,B) The enzyme LuxI generates AHL, a diffusible signaling molecule. The transcription factor LuxR, when bound to AHL, activates transcription of virulence genes at promoter pR. (A) LuxI-feedback system: LuxI is expressed from promoter pR, while LuxR is expressed from a constitutive promoter pX. (B) LuxR-feedback system: LuxR is expressed from promoter pR, while LuxI is expressed from a constitutive promoter pX. (C,D) Virulence gene expression as a function of cell density (computed from Eqs. 4, 5, 16) for: the wild type system (solid); with a LuxI inhibitor (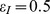; dot-dashed); with a LuxR non-competitive inhibitor (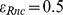; dashed); and with a LuxR competitive inhibitor (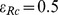; dotted). Positive feedback produces induction curves with stable upper and lower branches separated by an unstable middle branch. Cells which start off on the un-induced lower branch will jump to the highly-induced upper branch when their density crosses a critical threshold. (C) LuxI-feedback system. (D) LuxR-feedback system.

Gram-negative bacteria use a QS system mediated by diffusible signaling molecules of the acyl-homoserine lactone (AHL) family [12]. The mechanism of AHL QS was first elucidated in the marine bacterium *Vibrio fischeri* [35] (recently reclassified *Aliivibrio fischeri* [36]), but its molecular basis is conserved across several pathogenic and non-pathogenic bacterial species [37], [38]. AHLs are small organic molecules consisting of a homoserine ring and a variable species-specific acyl side chain [39]. AHL is synthesized from the precursor S-adenosylmethionine (SAM) by the enzyme LuxI [40], [41]. Low molecular weight AHLs are freely diffusible across the cell membrane, while high molecular weight ones are pumped [42], [43]. At high cell densities and therefore high AHL concentrations, AHL forms a complex with transcriptional regulator LuxR, which in turn activates expression at its cognate promoter pR [44]. In many bacterial species, for example the human pathogen *Pseudomonas aeruginosa* and the plant pathogen *Agrobacterium tumefaciens*, the LuxI gene itself is the under control of the LuxR-dependent promoter, forming a transcriptional positive-feedback loop [45] (Fig. 1A,B). Feedback might be essential to the functioning of QS systems, triggering a rapid onset of gene expression at a threshold cell density [46] (Fig. 1C,D).

We recently reported a comprehensive experimental characterization of *Vibrio fischeri* LuxI/LuxR quorum sensing molecules [46]. *V. fischeri* uses its QS system to regulate the expression of bioluminescence genes, but the virulence genes of many pathogens are regulated by analogous systems. Here we use biochemical parameters extracted from the *V. fischeri* experiments to build a molecular-level model of QS, and use this model to test the efficacy of combination drug therapies targeted against QS-regulated virulence genes. QS inhibitors exert their effects at multiple levels: the inhibition of AHL synthesis by LuxI; the degradation of AHL; the inhibition of AHL-LuxR complex formation; and the degradation of LuxR [17]–[21]. We examine each of these strategies individually and in combination. To understand the robustness of combination inhibitor therapies across diverse bacterial species, we test each strategy against a number of biochemical and transcriptional variants of the experimentally validated QS model.

We find that a combination of LuxI and LuxR non-competitive inhibitors act multiplicatively to inhibit virulence for a broad range of QS systems. In contrast, we find that LuxR competitive inhibitors act antagonistically with LuxI inhibitors, due to the weak activation of LuxR; in some conditions this can actually increase virulence. Both these results are somewhat surprising, and seem to arise due to the global structure of QS systems. Combination therapies must therefore be used with care, only once the most relevant drug combinations and molecular targets have been identified for each pathogenic species and infection context.

## Methods

### The Quorum Sensing Model

We first present a brief derivation of a quorum sensing model that has been developed in greater depth elsewhere [46]. Our model must take into account three types of dynamics: that of the cell population; that of the intracellular protein concentrations; and that of the diffusible signal. We imagine cells, each of mean volume *V_C_*, growing in a niche of total volume *V*. Let *ρ* represent the number-density of cells, exponentially growing with growth rate-constant *γ_C_*; we assume that the cell volume-fraction 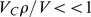. Let *Y_I_* and *Y_R_* represent the total intracellular concentrations of the enzyme LuxI and the transcription factor LuxR. Let *γ_I_* and *γ_R_* represent protein degradation rates, while *Q_I_* and *Q_R_* represent translation rates scaled by the respective decay rates. Let *φ* represent the concentration of AHL, whose intracellular and extracellular levels are equalized by rapid diffusion. AHL is synthesized by the enzyme LuxI at specific rate 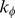; and it is removed from the niche, or equivalently degraded, with rate constant 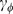. The protein transcription rates are as-yet-unspecified functions. The whole system may be described by the following set of coupled differential equations:
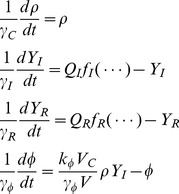
(1)


In realistic nutrient-limited conditions, intracellular protein levels equilibrate rapidly relative to the cell growth rate. For simplicity, we consider the situation in which AHL levels are modeled as the fastest variable, and cell density as the slowest, giving the following separation of timescales: 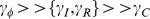. If we take the limit 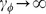, we obtain:
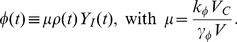
(2)


We can describe the binding of AHL to LuxR by a Hill equation with basal activity:
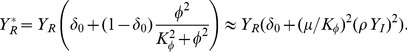
(3)


*Y_R_* is the total concentration of LuxR while Y_R_^*^ is the concentration of its active form; it is only the latter that drives transcription. We assume that AHL is in great excess over LuxR, so free AHL and total AHL levels are approximately equal. The Hill coefficient of 2 captures the fact that two molecules of AHL must bind in order to activate LuxR [47]. Homologs of LuxR are known to be able to bind DNA and activate transcription in the absence of AHL [48]. The basal level *δ*_0_ accounts for weak transcriptional activation by free LuxR. Our previous experiments suggest a non-zero value of *δ*_0_ [46]; however, the binding of free LuxR to DNA is not detectable when the protein is at nanomolar levels, so basal activity must be low. To derive Eq. 3, we have assumed 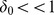, and 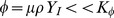. These assumptions have been validated in previous experimental measurements [46]. The latter condition, on AHL levels, arises because the affinity of active LuxR for its cognate promoter is in the nanomolar range, corresponding to a concentration of one protein per cell [47]. LuxR levels are significantly higher than this, so only a small fraction of LuxR need be bound to AHL in order to saturate transcription. Later we will derive a modified form of this equation to capture the effect of inhibitors.

We parameterize the rate of transcription at the virulence promoter by another Hill equation, where the term Y_R_^*^ represents total levels of active LuxR:
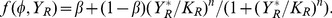
(4)


Here, we have defined the maximal transcription rate to be unity, without loss of generality. This simply means that we measure all other transcription rates relative to that of the virulence promoter. In particular, we assume that we are given a separate promoter whose transcription rate, under this normalization, is a constant value *α*. To describe feedback, we must substitute the functional form of Eq. 4 back into the dynamics of Eq. 1. Natural QS systems invariably place LuxI in transcriptional feedback [46]; for generality, we have considered both feedback topologies in our analysis (Fig. 1A,B). For LuxI feedback, we must set 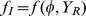 and 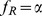; for LuxR feedback, we must set 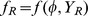 and 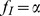. These options are represented by the following differential equations:
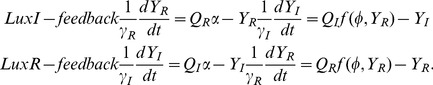
(5)


Assuming that cell density changes slowly we can find quasi-steady-state solutions to Eq. 5, which do not explicitly depend on the decay rates. The values of the remaining parameters (Table 1) are determined by fitting to expression data, as reported in Rai et al. [46]. Protein translation rates are specified in terms of fluorescence reporter units used in that study (FL), and cell densities are specified in terms of optical density at 600 nm (OD). Note that the steady states depend only on a few effective parameters which are algebraic combinations of the basic parameters. Taken together, these equations give the expression level of the virulence promoter as a function of cell density (Fig. 1C,D).

**Table 1:** Experimentally validated parameter values.

Parameter	Value	Dimension	Eq
*Q_I_*	8.382E3	*FL*	1,5
*Q_R_*	3.656E3	*FL*	1,5
*ρ*	0.05	*OD*	1,2,15
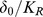	4.53E−4	*FL^−1^*	3,13,16
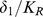	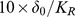	*FL^−1^*	13,16
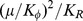	1.46E−6	*OD^−2^FL^−3^*	3,13,16
*n*	1.45	–	4
*β*	0.0282	–	4

### Modeling QS Inhibitors

We now describe a range of available inhibitory strategies (Table 2). Analogues of the LuxI substrate SAM, the precursor to AHL, act as competitive inhibitors of LuxI [49]. We can also consider using non-competitive inhibitors of LuxI and inhibitors that increase turnover of LuxI, as well as agents such as AHL lactonases which degrade AHL [50]. Since the levels of SAM are fixed, all three of these strategies effectively result in reduced levels of AHL for given levels of LuxI expression. Without loss of generality, we can model all these as we would a non-competitive inhibitor *I_nc_* of LuxI:
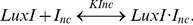
(6)


**Table:** **Table 2.** Classes of QS inhibitors.

Class	Example	Parameters
LuxI competitive inhibitor	SAM analogues	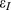
LuxI non-competitive inhibitor		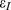
LuxI turnover		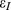
AHL turnover	AHL lactonases	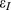
LuxR competitive inhibitor(basal induction)	AHL analogues	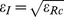
LuxR competitive inhibitor(moderate induction)	AHL analogues	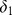 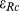 ,
LuxR competitive inhibitor(zero induction)	AHL analogues	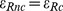
LuxR non-competitive inhibitor		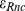
LuxR turnover	furanones	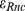

Assuming the bound complex cannot synthesize AHL, we find a modified version of Eq. 2:
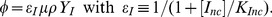
(7)


That is, the effect is simply a factor 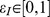 by which AHL levels are decreased; the higher the dose of inhibitors, the lower the level of 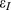.

A variety of natural products such as halogenated furanones have been found to bind LuxR and increase its turnover [51]. As we did with LuxI above, we can model these as non-competitive inhibitors *R_nc_* that reduce overall levels of LuxR (in all its forms):
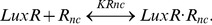
(8)


Total LuxR levels in Eq. 3 must therefore be modified by a factor:
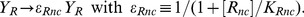
(9)


We have ignored here the possible complication that furanone binding might displace bound AHL. In general, synthetic AHL analogues act as competitive inhibitors of LuxR by binding the same pocket as AHL. The resulting complex often shows weak rather than zero transcriptional activity [52]. To model this effect, we let *φ* represent the concentration of AHL; but now we also consider the concentration of the AHL analogue, the competitive inhibitor *R_c_*. Assuming completely cooperative binding, we have the following reactions:
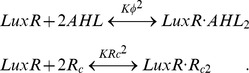
(10)


In terms of total LuxR levels *Y_R_*, the equilibrium concentrations of various forms of LuxR are given by:
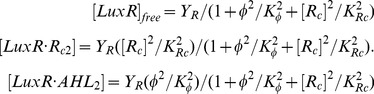
(11)


The complex 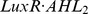 is the transcriptionally active form. However, both free LuxR as well as the 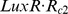 complex have probabilities 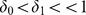 to be active [52]. The total amount of active LuxR is therefore given by:
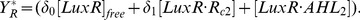
(12)


For simplicity, we assume that all three forms of active LuxR have the same affinity *K_R_* for DNA binding (Eq. 4). In the limit of 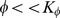 and 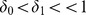, as in Eq. 3, we find:
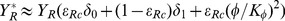
(13)where we have defined:



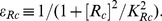
(14)For 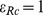 (when no inhibitor is present) this reduces to Eq. 3. The probability *δ*_1_ with which AHL analogues can themselves activate LuxR depends on the structure of the analogue, and can vary from 1.5 to 10 times the basal rate [52]; in our analysis we conservatively assume 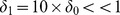. Lower values of *δ*_1_ reduce to simpler cases: if *δ*_1_ = 0 the 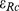 term factors out, and has the same effect as multiplying *Y_R_* by a factor 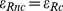 in Eq. 9; if *δ*_1_ =  *δ*_0_ the first two terms in Eq. 13 sum to a constant, and the remaining term has the same effect as multiplying *Y_I_* by a factor 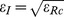 in Eq. 7 (Table 2).

Finally, we want to consider situations in which AHL itself is added externally. Suppose external AHL is provided at some constant concentration *φ*_0_; this simply amounts to:
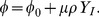
(15)


Taking Eqs. 7, 9, 13, 15 together, this gives a modified form of Eq. 3 in which the concentration of active LuxR is given by:
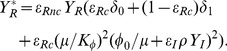
(16)


This can be substituted into Eq. 4 to obtain the final dynamics.

In our analysis, we will focus on the rate of expression at the virulence promoter at a fixed cell density *ρ* (Eq. 4; Fig. 1C,D). To state that inhibitors decrease virulence expression at some cell density is equivalent to saying that the threshold density for induction of virulence has increased. We calculate virulence expression as a function of the inhibitory parameters 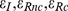, which vary from 1 (in the absence of inhibitors) to 0 (at high inhibitor concentrations). We set the remaining parameters to values shown in Table 1, unless otherwise mentioned. Notice that, due to transcriptional feedback and non-linearities, the effect of adding inhibitors in combination cannot be easily deduced from the form of Eq. 16.

## Results

### LuxR Activation

In deriving Eq. 16, The condition 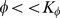 and 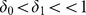 corresponds to an interesting situation: only a small fraction of LuxR is bound to AHL but all of this component is active; whereas the bulk of LuxR is free or bound to the analogue, but only small fractions of these components are active. Thus all three components contribute non-negligibly to transcriptionally competent LuxR. It is instructive to get some idea of the numbers, for the parameter values given in Table 1. There is some basal amount of active LuxR in the absence of AHL and its analogues. In the absence of the analogue, LuxI needs to be expressed at 0.125 times the unit transcription rate for AHL-induced LuxR to reach 9 times basal levels, so the total amount of active LuxR is 10 times basal levels. If LuxI is expressed at unit transcription rate, AHL-induced LuxR reaches 560 times basal levels; if LuxI is expressed at a tenth of this rate, AHL-induced LuxR is only 5.6 times basal levels. If the analogue is now added at high concentrations, analogue-induced LuxR is at 10 times basal levels independent of the AHL concentration (since we assume 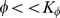 under physiological conditions). Thus, addition of analogue can either increase or decrease transcription, depending on the original level of AHL. The actual rate of transcription at the virulence promoter is determined by the value of 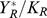 in Eq. 4; that is, it depends on the total amount of LuxR in the cell relative to its DNA binding affinity. If LuxR is expressed at unit rate, then in the absence of AHL and its analogues we will have 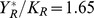, and the virulence promoter will already be at half-saturation; at high levels of the analogue we will have 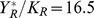, and the promoter will be fully induced. In the section below on ‘Robustness of inhibitory effects’, we discuss in detail what happens if total LuxR levels are much lower than this. The main message is that LuxR must be at relatively low levels, corresponding to 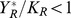, in order for the promoter to be sensitive to AHL.

### Effect of Individual Inhibitors

**Figure 2:**
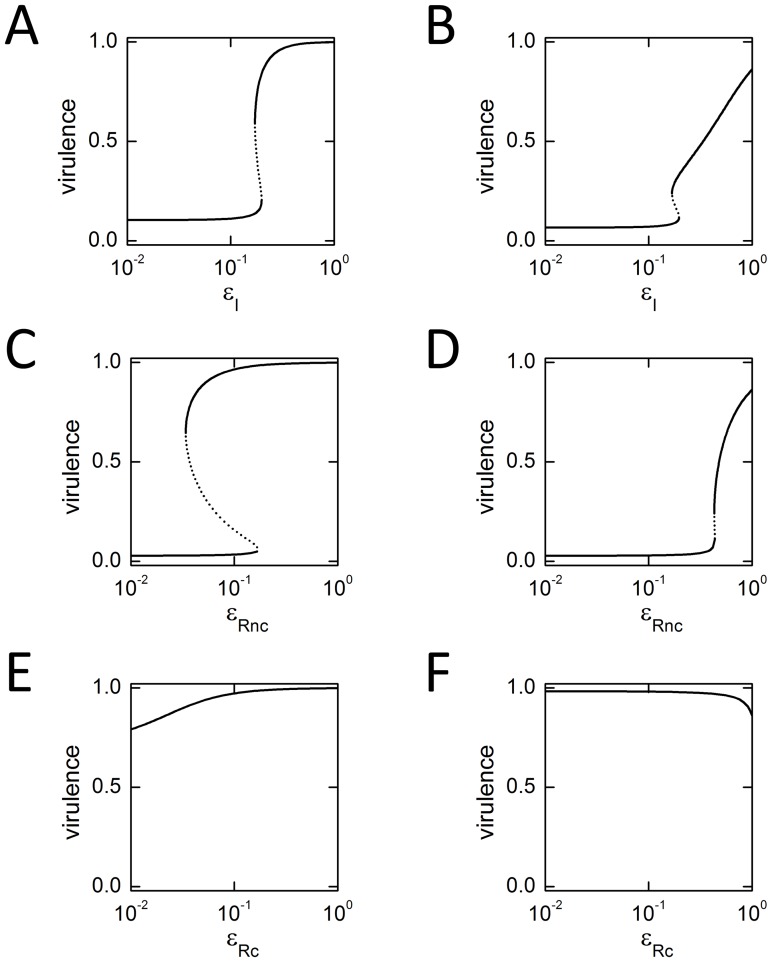
Effect of individual inhibitors. Each panel shows the steady-state expression level at the virulence promoter as a single inhibitor is varied (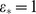 in the absence of inhibitors, 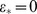 at high inhibitor levels). Parameter values are taken from Table 1. The *x*-axis is logarithmic. Due to positive feedback, the equations sometimes admit three solutions for a fixed level of inhibition: the upper and lower branches are stable (solid curves); the middle branch is unstable (dotted curve). (A,C,E) LuxI-feedback systems with *α* = 0.11. (B,D,F) LuxR-feedback systems with *α* = 0.05. (A,B) LuxI inhibitors (varying 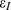). (C,D) LuxR non-competitive inhibitors (varying 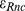). (E,F) LuxR competitive inhibitors (varying 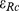).

We examined the effect of the three inhibitory strategies (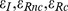) acting independently on the two possible feedback topologies (Fig. 2). In general, as expected, increased inhibition (reduced 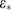 values) typically results in decreased virulence gene expression. Since both feedback loops have the potential for bistability, the inhibition curve sometimes has an upper and a lower branch, with the middle branch representing an unstable state (Fig. 2A–D). Since cells originally start in an induced state, the inhibition level 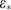 must be brought to a value where the upper branch vanishes before virulence is truly suppressed. In the LuxI-feedback case, this threshold is more easily reached using an inhibitor of LuxI rather than a non-competitive inhibitor of LuxR (the threshold value of 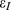 is closer to one in Fig. 2A compared to Fig. 2C). In the LuxR-feedback case the situation is reversed, with the non-competitive LuxR inhibitor more easily able to suppress virulence (the threshold value of 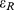 is closer to one in Fig. 2D compared to Fig. 2B). This contrast arises because a small initial decrease of the protein in feedback will loop around to produce further decreases, whereas this cascading effect does not occur for the protein outside of feedback. Note that inhibition to just 10% of original levels is sufficient in many cases to suppress virulence expression (Fig. 2A,B,D). Finally, we see that it is possible, under certain conditions, for LuxR competitive inhibitors to slightly increase virulence gene expression from the unperturbed state (Fig. 2F). As discussed in the previous section, this counter-intuitive result arises because our model allows for the fact that LuxR, when bound to competitive AHL analogues, is not completely inactive. If the unperturbed level of AHL is below some threshold, then the analogue actually has a positive influence. This effect is explored in more detail in the section below on ‘Robustness of inhibitory effects’.

### Effect of Inhibitors in Combination

**Figure 3:**
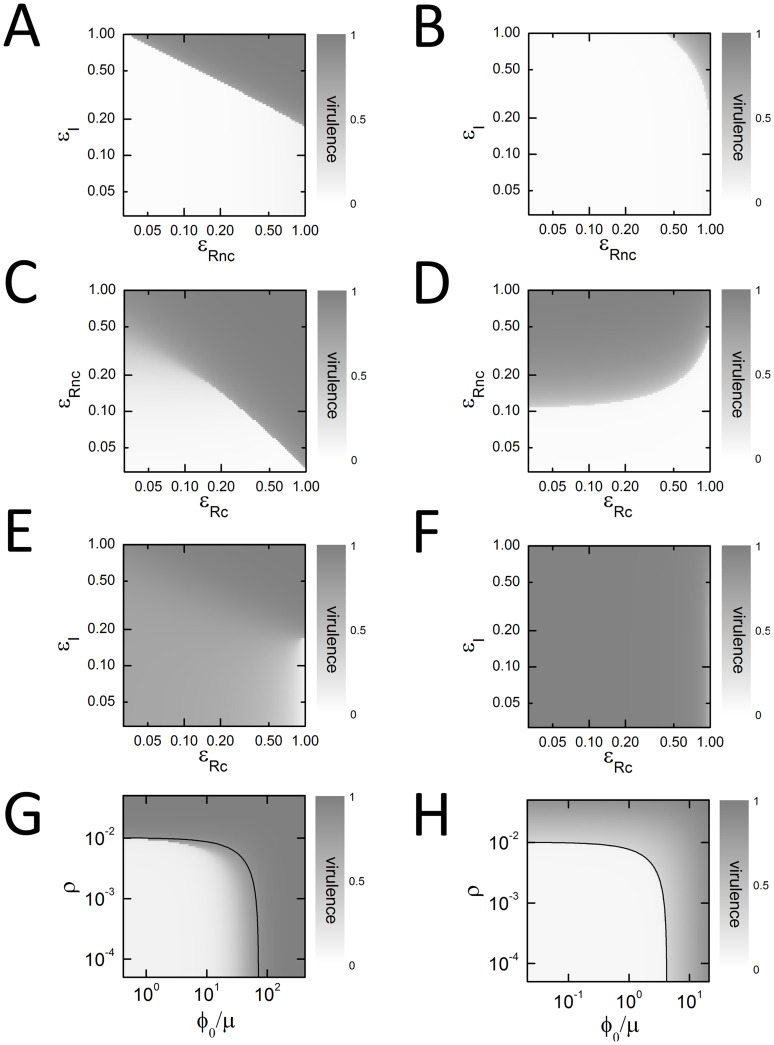
Effect of inhibitors in combination. Each panel shows the steady-state expression level at the virulence promoter as two inhibitors are varied. Parameter values are taken from Table 1. Both *x*- and *y*-axes are logarithmic. Virulence expression is represented by the shade, ranging from low virulence (light) to high virulence (dark). When two stable branches co-exist, the upper branch is shown (except in panels G,H, where the lower branch is shown). Sharp transitions represent bifurcation points where the upper branch vanishes. (A,C,E,G) LuxI-feedback systems with *α* = 0.11. (B,D,F,H) LuxR-feedback systems with *α* = 0.05. (A,B) LuxI inhibitors and LuxR non-competitive inhibitors. (C,D) LuxR non-competitive inhibitors and LuxR competitive inhibitors. (E,F) LuxI inhibitors and LuxR competitive inhibitors. (G,H) External AHL is varied along the *x*-axis, while cell density is varied along the *y*-axis. The dark curve shows the contour of constant total AHL from external and internal sources.

We next examined the effect of adding inhibitors in pairs. We can represent our results on a two-dimensional plot where some 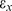 is varied on one axis, while some other 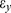 is varied along the other. In Figure 3, the shading represents the rate of expression at the virulence promoter, with darker values representing higher virulence levels. If two branches of solutions exist (as in Fig. 2A–D) we only show the expression on the upper branch. We occasionally see sharp transitions from dark to light, when the upper branch vanishes (Fig. 3A,B,D,H and part of Fig. 3C). But we also see smooth transitions from darker to lighter shades (Fig. 3E,G and part of Fig. 3C). These non-linearities are typical signatures of positive feedback.

We can understand the structure of these graphs as follows. The leading term of Eq. 16, seen as a function of *Y_I_* and *Y_R_*, has the form 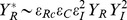. This suggests that, in the absence of feedback or basal activity terms, different inhibitors should act multiplicatively. That is, so long as the product 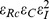 is held constant, the virulence expression level will be unaltered. The dependence of virulence on the product itself is typically highly non-linear; multiplicative behavior would therefore appear as a diagonal line of slope −1 or −1/2 separating dark and light regions on the log-log plots of Figure 3. If feedback or basal activity terms are strong, we will see deviations from this simple multiplicative behavior.

We find that LuxI inhibitors and LuxR non-competitive inhibitors act essentially multiplicatively, on both LuxI-feedback and LuxR-feedback systems (see Fig. 3A,B; though as we saw previously, the LuxR inhibitor is more potent for LuxR-feedback systems). The effect of LuxR competitive inhibitors is more subtle. LuxR competitive and non-competitive inhibitors can act multiplicatively over a certain range of inhibition (e.g. Fig. 3C). However, the typical case is that LuxR competitive inhibitors act antagonistically in combination with either LuxI inhibitors or LuxR non-competitive inhibitors, generating results that are worse than those using individual inhibitors (Fig. 3D,E,F).

### Effect of LuxR Agonists

In addition to inhibitory strategies, it has been suggested that cells driven to prematurely express virulence genes due to exposure to LuxR agonists are more likely to be cleared by host immune system [53]; such agonists would also be of benefit in systems where virulence is negatively regulated by QS [54]. This strategy is modeled by the addition of some concentration *φ*_0_ of external AHL (Eq. 16). If the goal is to trigger an immune response, the outcome can be measured by asking at what cell density *ρ* virulence genes are expressed, for varying levels of external AHL (Fig. 3G,H); the lower this density, the greater the chances of a successful clearance. For LuxI-feedback systems, we find that the system is smoothly induced at AHL levels below those required for autonomous induction, due to the effect of positive feedback (Fig. 3G). For LuxR-feedback systems, there is simply a constant threshold level of AHL required for induction; this can be achieved externally, internally, or by any combination of the two (Fig. 3H).

### Robustness of Inhibitory Effects

**Figure 4:**
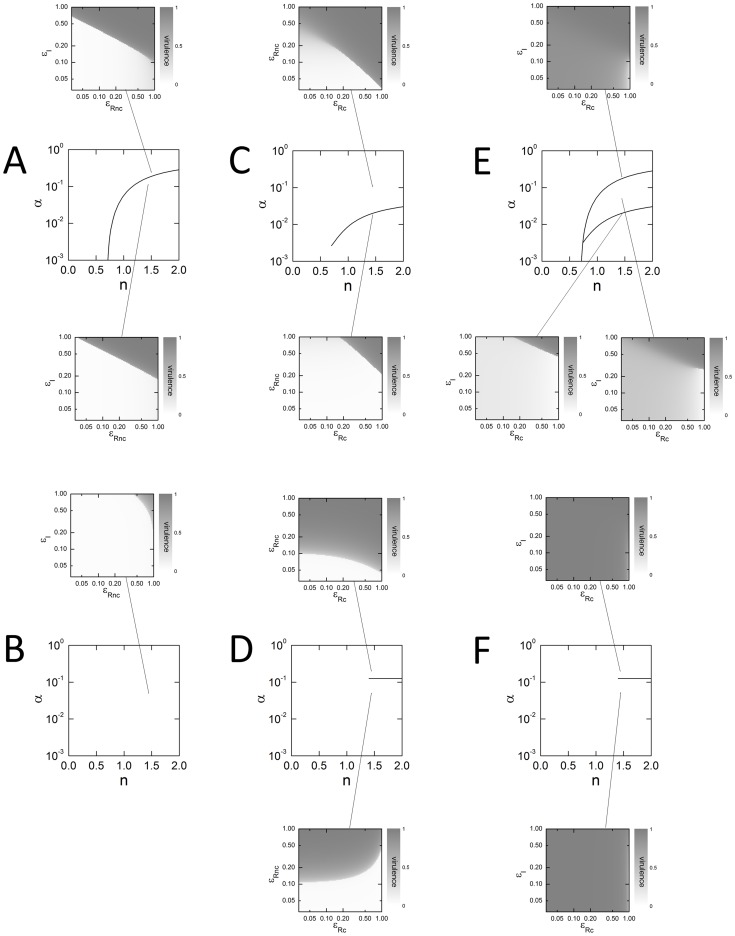
Dependence on parameters. We vary two key parameter values (keeping the rest fixed at the values shown in Table 1): *α*, the expression level of the constitutive promoter; and *n*, the Hill coefficient of LuxR-DNA binding. As we move through this space of parameters, the effect of inhibitor combinations will change. Dark curves show transitions between qualitatively different inhibitor effects: emergence of smooth or sharp transitions, or a change of curvature of the inhibitory boundary. Examples of different inhibitor effects are shown as 2-dimensional plots, as in Fig. 3, with light connecting lines indicating the parameter values that give rise to each plot. (A,C,E) LuxI-feedback systems. (B,D,F) LuxR-feedback systems. (A,B) LuxI inhibitors and LuxR non-competitive inhibitors. (C,D) LuxR non-competitive inhibitors and LuxR competitive inhibitors. (E,F) LuxI inhibitors and LuxR competitive inhibitors. (A) The two inhibitors act essentially multiplicatively. The response is abrupt for low values of *α* (below the curve) but is a mixture of smooth and abrupt for high values of *α* (above the curve). (B) The two inhibitors act multiplicatively, with no qualitative changes over the parameter range. The response is abrupt. (C) The two inhibitors show complicated interactions. For low values of competitive inhibition (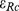 close to 1), the interaction with the non-competitive inhibitor is antagonistic. For higher values of competitive inhibition (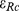 close to 0) the interaction with the non-competitive inhibitor is multiplicative. The dark curve in {*α*,*n*} space separates purely abrupt responses from a mixture of smooth and abrupt responses. (D) Above the value *α* = 0.125, both the LuxR competitive and non-competitive inhibitors act to suppress virulence. However, it is mainly the level of the LuxR non-competitive inhibitor which is important. Below the value *α* = 0.125, the competitive inhibitor acts antagonistically with the non-competitive inhibitor. For *n* >1.4 the response is abrupt; for *n* <1.4 the response is smooth. (E) The two inhibitors show similar interactions as in panel (C). The dark curves separate regions where the response is completely smooth (top), completely abrupt (bottom left) or a mixture of the two (bottom right). (F) Virulence expression is high over all inhibitor combinations and parameter values shown. The distinction between cooperative and antagonistic behavior is hardly visible.

All the results up to this point apply to the experimentally validated parameter set shown in Table 1. However, these parameters are likely to vary between the QS systems of different pathogens. To explore the influence of parameter variations, we calculated the effect of inhibitor combinations as two key parameters were varied (Fig. 4): *α*, the expression level of the protein outside of feedback (Eq. 5); and *n*, the Hill coefficient of LuxR-DNA binding (Eq. 4).

We find that the combination of LuxI inhibitors and LuxR non-competitive inhibitors act multiplicatively across a wide range of parameters, and for both LuxI-feedback as well as LuxR-feedback systems (sharp diagonal lines in Fig. 4A,D). This is a robust therapeutic strategy and can be applied without detailed biochemical knowledge of the underlying system.

As we saw previously, LuxR competitive inhibitors present a more complicated picture. The combination of LuxR competitive and non-competitive inhibitors can have many types of effects: essentially multiplicative (Fig. 4B, where lack of one inhibitor can be made up by surplus of another); canalizing (Fig. 4E, upper panel, where it is only the level of the non-competitive inhibitor which has a major effect); and even antagonistic (Fig. 4E, lower panel, where higher amounts of LuxR competitive inhibitor actually increase the levels of LuxR non-competitive inhibitor required to suppress virulence). The combination of LuxI inhibitors with LuxR competitive inhibitors performs worst of all the strategies tested here. In this case, overall levels of AHL tend to drop as the LuxI inhibitor is added. This makes the weakly activating effect of the LuxR competitive inhibitor all the more potent, leading to high levels of virulence across the entire range of inhibitor concentrations (Fig. 4C,F).

In assessing the effect of LuxR competitive inhibitors, the rule of thumb is as follows: LuxR competitive inhibitors will suppress virulence only if the AHL level in the absence of inhibitors is above some threshold. For the parameters we have used, this threshold corresponds to a LuxI transcription rate of 0.125 (as explained in the section Results: LuxR activation). For the LuxR-feedback system this can be seen as a horizontal line at *α* = 0.125 in Fig. 4E,F, independent of inhibitor levels. The condition is more complicated for LuxI-feedback systems, where the transition from virulence suppression to virulence activation by LuxR competitive inhibitors can happen at intermediate inhibitor levels (Fig. 4B). Of course, if the competitive inhibitor does not activate LuxR above basal levels (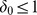), these considerations will not apply: the effect of the LuxR competitive inhibitor will then be analogous to that of a LuxI inhibitor, serving to decrease effective AHL levels.

## Discussion

QS inhibitors are promising alternatives to antibiotics, but there are still many steps on the path to their widespread use. It has been argued that pathogens targeted with QS inhibitors would be under weaker selective pressure to develop resistance, compared to the pressures induced by antibiotics [16]. However, the reality is more complex: in an infection context, individuals resistant to QS inhibition have a major advantage, and tend to be selected [26]–[28]. Combination drug therapies that target multiple molecules simultaneously would lower the rate at which such resistant individuals spontaneously arose. This motivated us to ask which QS targets would respond best to simultaneous inhibition. QS being implemented by a non-linear feedback system, the answer to such a question is far from obvious: it will vary from one pathogen to another, depending on the underlying feedback topology and biochemical parameter values. However, our analysis does produce some robust results.

We find that a combination of LuxI inhibitors and LuxR non-competitive inhibitors has the greatest capacity to suppress virulence, across a wide range of parameters. This strategy should be considered as the default: it can be applied without detailed knowledge of the pathogen’s QS system; moreover, since it targets two distinct molecules, the likelihood of spontaneous resistance is reduced. In contrast, LuxR competitive inhibitors should be used with care. These molecules tend to be AHL analogues with some weak capacity to activate LuxR. Though this capacity is much less than that of AHL itself when measured per molecule, the overall effect depends sensitively on AHL levels at the site of infection. Since physiological AHL concentrations tend to be low, competitive inhibitors in the form of AHL analogues can paradoxically increase virulence gene expression. Recently, structure-function studies have been used to design AHL analogues that completely block LuxR-DNA binding [55]. Such studies show that LuxR competitive inhibitors might yet find use.

New experiments can help improve the design of anti-virulence therapies at three levels. First: Any mathematical model such as ours is limited by the accuracy of the equations used, and of the parameter values they contain. Careful biochemical measurements can improve the predictive power of these models. Second: no mathematical model can account for the complications of real-world therapy. The true test of any strategy can only come from experimental studies on animal models, and ultimately from clinical trials. Third: our results should be understood in the context of a wider range of strategies, including the use of QS inhibitors in combination with antibiotics [56], as well as the stimulation of the host immune system. Research on pathogen biology will add to this list, revealing new and unexpected strategies. The lesson learned from nearly a century of experience with antibiotics is that pathogens present a moving target, and any single strategy is likely to be of use only for a limited time.
